# Retrospective analysis of CD4 trends at district levels in South Africa between 2013 and 2023

**DOI:** 10.4102/sajhivmed.v27i1.1772

**Published:** 2026-02-26

**Authors:** Naseem Cassim, Pedro da Silva, Deborah K. Glencross, Wendy S. Stevens, Lindi-Marie Coetzee

**Affiliations:** 1 Wits Diagnostic Innovation Hub, Faculty of Health Sciences, University of the Witwatersrand, Johannesburg, South Africa; 2 National Priority Programme, National Health Laboratory Service, Johannesburg, South Africa

**Keywords:** HIV, CD4, advanced HIV disease, laboratory data, district

## Abstract

**Background:**

South Africa has the largest HIV epidemic globally. Despite the scale-up of antiretroviral therapy, people living with HIV still present with CD4 ≤ 200 cells/µL because of possible treatment interruptions and/or late presentation.

**Objectives:**

This study assessed the proportion of CD4 specimens with counts ≤ 200 cells/µL by health district, comparing 2013 to 2023.

**Method:**

This cross-sectional study analysed laboratory data. Annual test volumes, median CD4 and the percentage of specimens with a count ≤ 200 cells/µL were reported. The difference in the median CD4 between 2013 and 2023 was calculated. No de-duplication was possible because of the absence of unique identifiers.

**Results:**

Data are reported for 5 821 932 specimens, with a significant decline in absolute numbers, while the percentage of specimens with a count ≤ 200 cells/µL declined by 0.4%. The median CD4 increased from 396 cells/µL in 2013 to 465 cells/µL by 2023. Between 2013 and 2023, eight districts showed a decrease in the median CD4, while 44 reported an increase ranging from 2 cells/µL (Sedibeng) to 192 cells/µL (uThukela). A percentage point reduction for counts ≤ 200 cells/µL between 2013 and 2023 was reported for 23 districts (44.2%), while 29 (55.8%) showed a percentage point increase.

**Conclusion:**

Despite an upward trend in the national specimen-level CD4 count median over time, unacceptable levels of CD4 counts ≤ 200 cells/µL persisted. The analysis provides important insight into district burden of advanced HIV disease which warrants national attention and further investigation.

**What this study adds:** This study provides important insights into the percentage of specimens with a count ≤ 200 cells/µL at district level, masked by overall national and provincial proportions. Specific districts in four provinces were identified that warrant further investigation and intervention.

## Introduction

South Africa has the world’s largest HIV epidemic globally, with 7.7 million people living with HIV (PLHIV) in 2023.^1,2^ An HIV prevalence of 12.7% was reported in 2023.^3^ Several challenges remain, such as gaps in antiretroviral treatment (ART) coverage and sub-optimal virological suppression.^1,3,4,5^ Provinces such as KwaZulu-Natal and Mpumalanga experience hyper-epidemics, with prevalence exceeding 15%.^3^ Antiretroviral treatment coverage has increased substantially, reaching 77% of PLHIV by 2023.^1^ Data from the Thembisa model showed that between 2013 and 2023, ART coverage increased by 35.6% at the national level, while the number of new HIV infections decreased fivefold.^6^ In 2022, among PLHIV aged 15 years and older, 90% were aware of their status, 91% were on ART, and 94% were virally suppressed.^3^

Despite improvements in ART coverage and viral suppression, levels of advanced HIV disease (AHD) remain a concern.^7,8,9^ Advanced HIV disease is defined as a CD4 cell count < 200 cells/µL or WHO clinical stage 3 or 4.^10^ Reports suggest that almost 30% of people that initiate ART are either ART-naïve patients or re-entering after a period of disengagement.^9^ Carmona et al. reported that, between 2005 and 2011, the proportion of PLHIV aged ≥ 15 years entering care with a first CD4 count < 200 cells/µL declined from 46.8% to 35.6%.^7^ However, between 2011 and 2016, the proportion of patients entering care with CD4 counts < 200 cells/µL remained relatively unchanged, at 32.9%.^7^ A subsequent study reported that between 2017 and 2023, the percentage of specimens with a count ≤ 200 cells/µL ranged from 19.5% and 20.8%, despite a consistent increase in the median CD4.^8^ Between 2013 and 2023, the percentage of specimens with a CD4 count ≤ 200 cells/µL decreased minimally, by 0.3%.^8^ Of more notable concern is the increase in the percentage of specimens with a count ≤ 200 cells/µL between 2013 and 2023 in the Western Cape province.^8^

Re-entry into care by patients, after being on ART, at an advanced stage of HIV disease is also an important consideration.^9^ It has been reported that many PLHIV start and stop ART multiple times in a cascade through which they can re-enter HIV care.^11,12^ A Western Cape observational cohort study reported that 10% of PLHIV linked to HIV care disengaged after early ART initiation, and a further 12% disengaged after long-term ART.^2,13^ Similarly, Osler et al. reported that in 2016, across 10 sites, 51.8% of the PLHIV with first CD4 < 50 cells/µL were ART-experienced.^14^ Furthermore, 76% could be confirmed to either be off ART or to have had viraemia at the time of the index CD4 test.^14^ This highlights the dual challenges of re-entry into care as well as ART-experienced PLHIV presenting with AHD.^9,14^

Substantial local data have been generated and reported on the burden of PLHIV who have a CD4 count ≤ 200 cells/µL at the national and provincial levels, with limited data for districts.^1,8,15,16,17,18^

### Objective

The objective of this study was to conduct an evaluation of the proportion of CD4 specimens with counts ≤ 200 cells/µL across 52 metropolitan and municipal health districts in South Africa, comparing data from 2013 and 2023.

## Research methods and design

### Context

The public-sector National Health Laboratory Service (NHLS) serves over 80% of the South African (largely indigent) population.^19^ For the study period, CD4 testing was provided for using the following cytometry platforms supplied by Beckman Coulter (Miami, Florida, United States): (1) XL-MCL, (2) FC500 MPL/CellMek, and (3) Aquios CL. All CD4 data are collected via the laboratory information system (LIS) and collated into the NHLS Corporate Data Warehouse (CDW).^19,20^ Relevant demographic data, provided on paper-based hand-written sample request forms and captured onto the LIS, are used to identify individual specimen-level data points. A hub-and-spoke laboratory network offers both decentralised and centralised CD4 testing using a tiered delivery approach that ensures nationwide coverage of laboratory services across all levels of care, from busy metropolitan centres to hard-to-reach rural districts.^21^ Strict electronic gate keeping (EGK), which is implemented using a rule-based system in the LIS, prevents the unnecessary retesting of specimens within a specific time frame.^22^ In addition, local guidelines advise CD4 testing at specific timepoints and, based on this, there should not be more than one test performed on a single patient in a specified time. Therefore, EGK aims to limit retesting within the specified time interval in line with local guidelines.^23,24^

### Study design

A cross-sectional study design was used to analyse specimen-level CD4 laboratory data for calendar years 2013 and 2023 at the health district level. Because of the absence of unique identifiers on individual patient data records, it was not possible to de-duplicate data.^25,26^

### Data preparation

The data extract was provided by the NHLS CDW data repository, and included the following variables: (1) episode number, (2) result review date, (3) province, (4) health district, and (5) absolute CD4 count. The year and month were extracted from the result review date. The absolute CD4 count was categorised as ≤ 200 cells/µL and > 200 cells/µL. Data were limited to the nine provinces and 52 health districts reported in the District Health Information System organisational hierarchy.^27^ The datasets were prepared and analysed using SAS 9.4 (SAS Institute, Cary, North Carolina, United States) and Stata SE (Stata Corporation, College Station, Texas, United States). Province and district naming conventions conformed to the descriptions used by the South African Municipal Demarcation Board (MDB) shapefile.^28^ Chloropleth maps were created using ArcGIS (Environmental Systems Research Institute, Redlands, California, United States). A choropleth is a form of statistical thematic mapping that employs pseudocolour to visually represent aggregated geographic data.^29^ The choropleth colouration corresponds to a specific metric of a spatially defined unit.^29^ Spatial files for health districts were obtained from the MDB.^28^ Health district population estimates for 2013 and 2023 were obtained from Statistics South Africa to report the rate per 100 000 population,^30^ as shown in Equation 1:


$$\text{Rate per }100\;000\text{ population with a count}\leq 200\text{ cell}/\mu \text{L}=\left(\frac{Number\;of\;specimens\;with\;a\;CD4\leq 200}{Health\;District\;Population}\times 100\;000\right)$$ [Eqn 1]

The changes between 2013 and 2023 were also reported.

### Statistical analysis

The percentage of specimens with a count ≤ 200 cells/µL was reported by health district. The difference in the median CD4 between 2013 and 2023 was analysed by district, and the number of districts where the percentage of specimens with a count ≤ 200 cells/µL exceeded the national value was reported by province. Choropleth maps reported the percentage of specimens with a count ≤ 200 cells/µL, using the following buckets/categories with colours as indicated in brackets: (1) 6.6% – 11.9% (dark green), (2) 11.7% – 16.7% (light green), (3) 16.8% – 21.8% (yellow), (4) 21.9% – 26.9% (orange), and (5) 27.0% – 31.7% (red). The percentage change for specimens with a count ≤ 200 cells/µL between 2013 and 2023 was reported as a bar chart, with findings reported for each province. The data were analysed for districts with a percentage point increase between 2013 and 2023, reporting the number of specimens with a count ≤ 200 cells/µL in 2023 to identify districts with a higher burden. For this analysis, data were sorted in descending order by the number of specimens with a count ≤ 200 cells/µL, while the percentage of specimens with a count ≤ 200 cells/µL was reported as a line chart. The change in the rate per 100 000 population for a count ≤ 200 cells/µL between 2013 and 2023 was also reported.

### Ethical considerations

Ethical clearance for this study was obtained from the University of the Witwatersrand Human Research Ethics Committee (reference number: M220163). Anonymised secondary laboratory data were used.

## Results

Data are reported for 5 821 932 CD4 specimens (Table 1). The test volumes declined from 3 685 032 in 2013 to 2 136 900 by 2023. There was a decline in both absolute numbers of specimens with a CD4 count ≤ 200 cells/µL between 2013 (*n* = 762 868) and 2023 (*n* = 435 753), as well as the percentage (20.7% in 2013 to 20.3% by 2023). The national median CD4 was 396 cells/µL (interquartile range [IQR]: 233–581) in 2013, compared to 465 cells/µL (IQR: 243–706) by 2023, showing an increase of the median CD4 count by 69 cells/µL over 10 years.

**TABLE 1:** Health district analysis of the median CD4 and percentage of specimens with a count of ≤ 200 cells/μL by health district in 2013 and 2023.

Province district	Year											≤ 200 cells/μL Δ (%)
	2013		2023		Median Δ	2013			2023			
	Median	IQR	Median	IQR		Volume	≤ 200 cells/μL		Volume	≤ 200 cells/μL		
							*N*	%		*N*	%	
EC Alfred Nzo	396	234–586	492	274–719	96	48 571	10 004	20.6	26 535	4742	17.9	−2.7
EC Amathole	412	247–597	419	207–664	7	56 349	10 720	19.0	25 246	6132	24.3	5.3
EC Buffalo City	389	216–585	400	196–650	11	42 848	9815	22.9	37 565	9619	25.6	2.7
EC Chris Hani	381	221–560	392	196–628	11	58 975	13 006	22.1	26 588	6799	25.6	3.5
EC Joe Gqabi	376	215–563	452	236–686	76	25 946	5975	23.0	16 860	3503	20.8	−2.3
EC Nelson Mandela Bay	371	205–560	382	179–636	11	59 053	14 410	24.4	47 358	13 132	27.7	3.3
EC O R Tambo	398	236–584	421	217–659	23	97 472	19 884	20.4	42 228	9800	23.2	2.8
EC Sarah Baartman	403	239–596	390	185–642	−13	21 594	4273	19.8	20 379	5520	27.1	7.3
FS Fezile Dabi	370	211–544	400	194–634	30	36 257	8533	23.5	16 941	4355	25.7	2.2
FS Lejweleputswa	371	221–547	390	199–619	19	53 377	11 756	22.0	18 246	4604	25.2	3.2
FS Mangaung	386	222–572	415	202–668	29	58 601	12 958	22.1	34 051	8438	24.8	2.7
FS Thabo Mofutsanyana	370	220–540	460	238–692	90	62 993	13 948	22.1	28 295	5909	20.9	−1.3
FS Xhariep	382	220–570	467	242–712	85	7393	1638	22.2	5345	1092	20.4	−1.7
GP City of Johannesburg	370	206–551	409	212–639	39	293 812	71 349	24.3	190 815	44 964	23.6	−0.7
GP City of Tshwane	396	223–591	452	217–708	56	167 876	37 372	22.3	89 066	20 745	23.3	1.0
GP Ekurhuleni	369	210–547	373	182–609	4	223 713	53 013	23.7	102 301	28 140	27.5	3.8
GP Sedibeng	382	213–567	384	186–627	2	67 090	15 689	23.4	29 636	8035	27.1	3.7
GP West Rand	366	206–543	399	199–633	33	68 508	16 654	24.3	34 035	8560	25.2	0.8
KZN Amajuba	378	224–549	515	295–746	137	50 781	11 011	21.7	31 940	5087	15.9	−5.8
KZN eThekwini	407	254–584	504	284–738	97	346 417	61 427	17.7	178 559	29 706	16.6	−1.1
KZN Harry Gwala	401	247–581	547	293–823	146	33 287	6333	19.0	14 336	2383	16.6	−2.4
KZN iLembe	422	268–595	523	292–761	101	61 565	10 140	16.5	34 275	5521	16.1	−0.4
KZN King Cetshwayo	428	262–621	583	367–804	155	76 916	13 350	17.4	74 874	8252	11.0	−6.3
KZN Ugu	408	249–589	567	326–824	159	93 129	17 424	18.7	37 391	5307	14.2	−4.5
KZN uMgungundlovu	418	260–600	568	346–797	150	119 769	20 923	17.5	71 128	8851	12.4	−5.0
KZN uMkhanyakude	504	329–716	576	381–776	72	71 954	8590	11.9	53 721	5125	9.5	−2.4
KZN Umzinyathi	407	247–588	575	374–784	168	49 696	9 394	18.9	41 696	4200	10.1	−8.8
KZN uThukela	425	263–618	617	425–825	192	72 169	12 336	17.1	81 685	5429	6.6	−10.4
KZN Zululand	396	240–573	539	334–756	143	93 607	18 402	19.7	43 160	5551	12.9	−6.8
LP Capricorn	374	201–570	479	253–730	105	63 418	15 773	24.9	41 440	8111	19.6	−5.3
LP Mopani	370	214–548	391	187–635	21	70 100	16 171	23.1	23 369	6208	26.6	3.5
LP Sekhukhune	376	202–571	349	152–615	−27	42 475	10 543	24.8	17 121	5393	31.5	6.7
LP Vhembe	364	203–552	435	210–681	71	55 159	13 624	24.7	29 684	7122	24.0	−0.7
LP Waterberg	383	213–578	474	240–735	91	43 936	10 264	23.4	30 973	6465	20.9	−2.5
MP Ehlanzeni	420	248–614	447	235–692	28	178 774	34 166	19.1	66 723	14 041	21.0	1.9
MP Gert Sibande	397	233–582	548	338–770	151	95 763	19 892	20.8	73 112	9372	12.8	−8.0
MP Nkangala	378	218–556	390	191–635	12	76 664	17 311	22.6	36 804	9650	26.2	3.6
NC Frances Baard	424	252–630	477	247–730	53	29 236	5525	18.9	22 896	4624	20.2	1.3
NC JT Gaetsewe	396	232–581	477	269–702	81	13 459	2843	21.1	14 964	2625	17.5	−3.6
NC Namakwa	404	235–597	466	227–727	62	2566	516	20.1	2783	621	22.3	2.2
NC Pixley Ka Seme	403	245–592	470	249–715	67	12 571	2398	19.1	9002	1739	19.3	0.2
NC ZF Mgcawu	414	248–615	393	197–644	−22	9716	1829	18.8	9078	2306	25.4	6.6
NW Bojanala Platinum	386	221–574	402	201–650	16	92 853	20 783	22.4	43 782	10 900	24.9	2.5
NW Dr Kenneth Kaunda	396	222–596	417	213–664	21	65 673	14 631	22.3	36 911	8622	23.4	1.1
NW Dr RS Mompati	406	242–592	497	276–735	91	32 747	6433	19.6	27 786	4757	17.1	−2.5
NW NMi Molema	389	228–570	408	198–665	19	55 819	11 823	21.2	23 283	5877	25.2	4.1
WC Cape Winelands	425	263–619	344	162–599	−81	4263	724	17.0	21 100	6 690	31.7	14.7
WC Central Karoo	371	210–565	451	239–684	80	2085	486	23.3	2546	531	20.9	−2.5
WC City of Cape Town	424	266–598	391	189–632	−33	184 258	30 944	16.8	113 167	30 244	26.7	9.9
WC Garden Route	409	258–580	362	176–611	−47	20 241	3554	17.6	18 532	5350	28.9	11.3
WC Overberg	417	263–595	344	163–602	−73	7920	1324	16.7	7014	2209	31.5	14.8
WC West Coast	417	261–599	392	189–645	−25	5618	984	17.5	10 575	2795	26.4	8.9

**National**	**396**	**233–581**	**465**	**243–706**	**69**	**3 685 032**	**762 868**	**20.7**	**2 136 900**	**435 753**	**20.4**	**−0.3**

Dr, doctor; OR, Oliver Reginald; ZF, Zwelentlanga Fatman; RS, Ruth Segomotsi; JT, John Taolo; NM, Ngaka Modiri; EC, Eastern Cape; FS, Free State; GP, Gauteng; KZN, KwaZulu-Natal; LP, Limpopo; MP, Mpumalanga; NW, North West; NC, Northern Cape; WC, Western Cape; IQR, interquartile range; Δ, difference.

### Health district median CD4 analysis

In 2013, the health district median CD4 ranged from 364 cells/µL (IQR: 203–552) in Vhembe, to 504 cells/µL (IQR: 329–716) for uMkhanyakude (Table 1). For 2023, the median CD4 ranged from 344 cells/µL (IQR: 162–599) in Overberg to 617 cells/µL (IQR: 425–825) in uThukela. A wide variation in the median CD4 was noted in 2013 by district, ranging from 364 cells/µL (Vhembe) to 503 cells/µL (uMkhanyakude). A range of 344 cells/µL (Cape Winelands) to 617 cells/µL (uThukela) was reported for 2023. The change in the median CD4 between 2013 and 2023 ranged from –81 cells/µL (Cape Winelands) to 192 cells/µL (uThukela).

### Health district analysis: 2013

**FIGURE 1:**
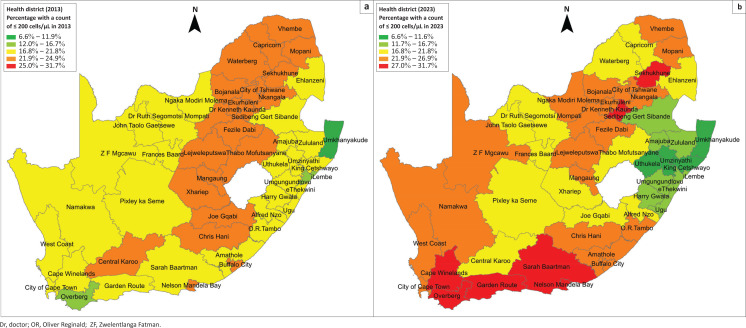
Chloropleth maps reporting the percentage of CD4 specimens with a count of ≤ 200 cells/μL by health district in 2013 (a) and 2023 (b) in South Africa. The map is categorised into five buckets as follows: (1) 6.6% – 11.9%, (2) 11.7% – 16.7%, (3) 16.8% – 21.8%, (4) 21.9% – 26.9%, and (5) 27.0% – 31.7%.

Only three districts (5.8%) reported a percentage of specimens with a count ≤ 200 cells/µL ≤ 16.7% in 2013 (Umkhanyakude [11.9%], iLembe [16.5%], and Overberg [16.7%]) (Figure 1). There were 23/52 (44.2%) districts that reported a percentage of specimens with a count ≤ 200 cells/µL in the 21.9% – 26.9% category, distributed as follows: (1) Free State (*n* = 5/5), (2) Gauteng (*n* = 5/5), (3) Limpopo (*n* = 5/5), (4) Eastern Cape (*n* = 4/8), (5) North West (*n* = 2/4), (6) Western Cape (*n* = 1/6), and (7) Mpumalanga (*n* = 1/3). There were no districts in the 21.9% – 24.9% category in the KwaZulu-Natal and Northern Cape provinces. The remaining districts reported a percentage of specimens with a count ≤ 200 cells/µL in the 16.8% – 21.8% category. No districts were allocated to the 27.0% – 31.7% category.

### Health district analysis: 2023

There were eight districts (15.4%) where the percentage of specimens with a count ≤ 200 cells/µL was classified in the 27.0% – 31.7% bucket, namely: (1) Western Cape: Cape Winelands, Garden Route, and Overberg, (2) Eastern Cape: Nelson Mandela Bay, and Sarah Baartman, (3) Gauteng: Ekurhuleni, and Sedibeng, and (4) Limpopo: Sekhukhune (Figure 1). On the opposite spectrum, there were 12 districts (23.0%) with a percentage of specimens with a count ≤ 200 cells/µL classified in the 6% – 11.9% and 11.7% – 16.7% buckets.

### Health district percentage point change for counts ≤ 200 cells/µL between 2013 and 2023

**FIGURE 2:**
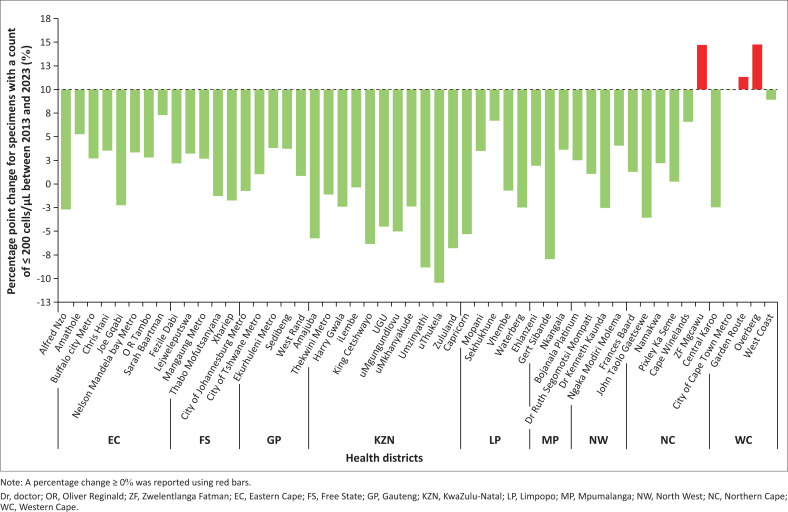
Analysis of the percentage point change for specimens with a count of ≤ 200 cells/µL between 2013 and 2023 by health district, grouped by province.

Of 52 districts, 23 (44.2%) showed a reduction, while 29 (55.8%) reported an increase. All 11 KwaZulu-Natal districts saw declines (up to –10.4% in uThukela). In the Eastern Cape, Alfred Nzo and Joe Gqabi reported percentage point reductions over two points; others increased by up to 7.3%. The Free State had two declining districts, while others rose to 3.2%. Only Johannesburg declined in Gauteng (−0.7%); other districts increased up to 3.8% (Figure 2). In Limpopo, three districts declined (lowest: −2.5% in Waterberg), while Mopani rose by 3.5% and Sekhukhune by 6.7%. Mpumalanga’s Gert Sibanda declined (−8.0%), while two others increased. In the North West, only Dr Ruth Segomotsi Mompati declined (−2.5%). Northern Cape’s John Taolo Gaetsewe dropped (−3.6%), with others rising to 6.6%. In the Western Cape, only Central Karoo declined (−2.5%); others rose sharply, up to 14.8% in Overberg.

### Health district analysis: Rate per 100 000 population

**FIGURE 3:**
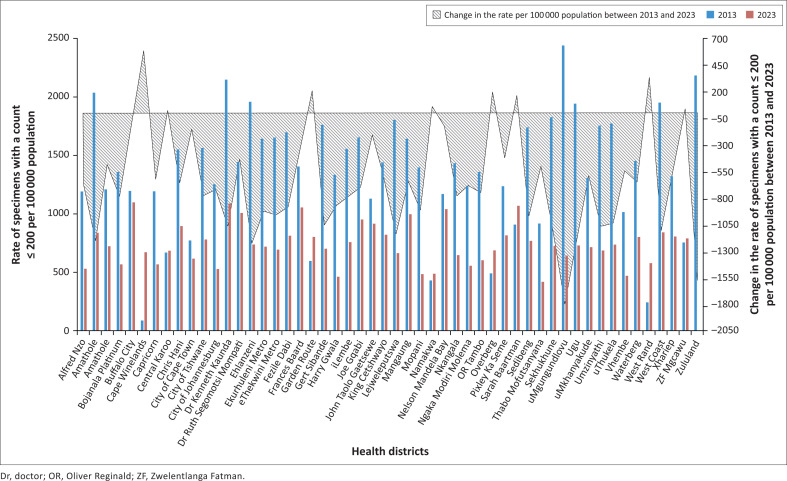
Analysis of the rate of specimens with a count of ≤ 200 cells/µL per 100 000 population in 2013 and 2023, with the change reported on the secondary y-axis.

In 2013, the district rate per 100 000 population for specimens with a count ≤ 200 cells/µL ranged from 87 (Cape Winelands) to 2441 (Ugu), compared to a national value of 1414 (Figure 3). By 2023, the national rate per 100 000 population had decreased to 701 (a reduction of 713 from 2013). The district rate per 100 000 population for a count ≤ 200 cells/µL in 2023 ranged from 414 (Sekhukhune) to 1096 (Buffalo City). The change in the district rate per 100 000 population for a count ≤ 200 cells/µL between 2013 and 2023 ranged from –1797 (Ugu) to 584 (Cape Winelands). The districts where the biggest change in the rate per 100 000 population for a count ≤ 200 cells/µL between 2013 and 2023 was noted were Cape Winelands, Garden Route, Overberg, Sarah Baartman, and West Coast.

### Health district analysis: Percentage point increase between 2013 and 2023

**FIGURE 4:**
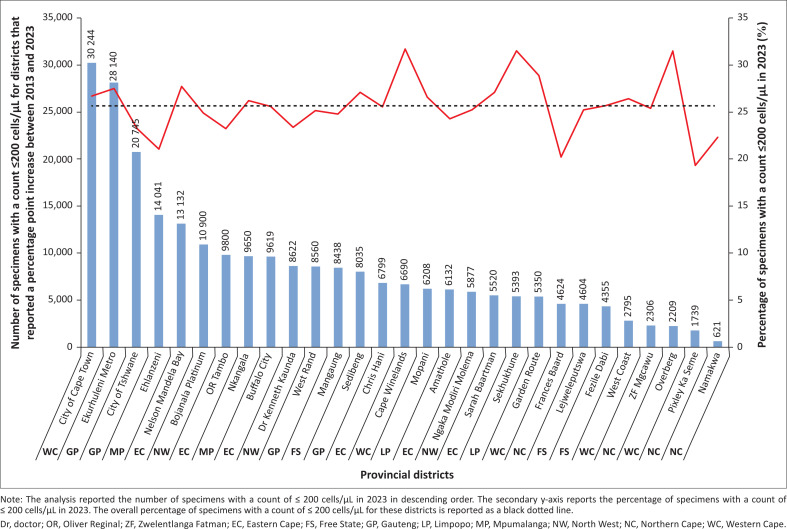
Analysis for districts where the percentage point change for specimens with a count of ≤ 200 cells/µL between 2013 and 2023 decreased.

Among the 29 districts (55.8%) reporting a percentage point increase, 17 contributed the most specimens with CD4 counts ≤ 200 cells/µL (Figure 4). Thirteen of these districts (44.8%) exceeded this threshold, with the highest percentage point increases observed in Cape Winelands, City of Cape Town, and Ekurhuleni Metro. Other districts with elevated highest percentage point increases included Fezile Dabi, Garden Route, Mopani, Nelson Mandela Bay, Nkangala, Overberg, Sarah Baartman, Sedibeng, Sekhukhune, and West Coast.

## Discussion

This study aimed to evaluate the proportion of CD4 specimens with counts ≤ 200 cells/µL across 52 health districts in South Africa, comparing data from 2013 and 2023. Our findings reveal a slight improvement in the percentage of specimens with counts ≤ 200 cells/µL. However, a substantial increase in the median CD4 count was noted. The improvement in the percentage of specimens with counts ≤ 200 cells/µL as well as the median CD4 was predominantly noted in the KwaZulu-Natal province, followed by selected districts in the Eastern Cape, Limpopo, Mpumalanga, North West, and the Northern Cape provinces. Districts in the Western Cape (Cape Winelands, Overberg, and Garden Route), Eastern Cape (Sarah Baartman, and Nelson Mandela Bay), Gauteng (Ekurhuleni, and Sedibeng) and Limpopo (Sekhukhune) provinces, reported proportions of specimens with counts ≤ 200 cells/µL exceeding 27% by 2023. This is high compared to national values of around 20% in both 2013 and 2023. This was supported by the decrease observed in the median CD4 noted between 2013 and 2023 in these eight districts.

The increase in the percentage of specimens with counts ≤ 200 cells/µL, specifically in the Western Cape, is consistent with a local analysis of CD4 data for the same period.^8^ What is surprising is that the district analysis in this study reported a percentage of specimens with counts ≤ 200 cells/µL 27% also being seen in other provinces, such as the Eastern Cape, Gauteng, and Limpopo provinces. This finding was not expected and could in part be explained by lower ART coverage rates, compared to a national ART coverage of 76.6% identified by the Thembisa model.^31^ In 2023, ART coverage rates for Western Cape (73.2%), Eastern Cape (71.6%), Gauteng (72.1%), and Limpopo (71.1%) provinces were lower than other provinces.^8^ The KwaZulu-Natal province, with the lowest percentage of specimens with counts ≤ 200 cells/µL, reported an ART coverage of 83.4%.^6^ Despite the national increase in ART coverage by 35.6% between 2013 and 2023, selected districts reported a high level of the percentage of specimens with counts ≤ 200 cells/µL, that could in part be explained by lower ART coverage.^6^

There are multiple factors that could contribute to the differences noted in the percentage of specimens with counts ≤ 200 cells/µL at the district level. One possible explanation may relate to some districts having a much higher HIV/tuberculosis co-infection rate, reported to exceed 50%.^32^ An analysis of 2023 laboratory data for the percentage of specimens with counts ≤ 200 cells/µL and the *Mycobacterium tuberculosis* complex detection rates at the district level, reported a correlation coefficient of 0.62.^33^ This indicates that HIV/tuberculosis coinfection might explain some of the differences in the percentage of specimens with counts ≤ 200 cells/µL.^33^ This was repeated for reflexed cryptococcal antigenaemia detection rates offered for counts < 100 cells/µL, surprisingly reporting a negative correlation coefficient of −0.209.^33^ Cryptococcal antigenaemia is a common and clinically significant opportunistic infection in individuals with AHD, often serving as a precursor to cryptococcal meningitis, and associated with increased morbidity and mortality in the absence of timely screening and pre-emptive antifungal therapy.^23,24^ However, factors such as other co-infections are outside the scope of this study, and may have played a contributing role.^10,24^

Additionally, socioeconomic factors, as well as access to healthcare services, may also play a role.^34,35^ Delays in diagnosis and treatment may also contribute, including poor communication and long waiting times at public health facilities that could discourage patients from seeking timely care.^36^ Integration of local clinical and laboratory data is urgently needed, specifically to understand the underlying reasons for the worrying trends documented here in the Cape Winelands, Overberg, Garden Route, Sarah Baartman, and Sekhukhune districts, where a median CD4 decrease is reported in this study between 2013 and 2023. Importantly, integration of this database would enable further work to identify what percentage of testing was done for ART-naïve patients versus patients re-entering care. Furthermore, the disaggregation of data by facility type would help to elucidate where these patients with a count ≤ 200 cells/µL are presenting for care (at hospitals or primary healthcare facilities). Finally, a geospatial analysis should be conducted using facility coordinates to identify hotspots in these districts. These findings could be used to focus programmatic interventions.

Despite advancements in the accessibility of HIV services as well as improved ART coverage, the wide gap in the median CD4 at the district level across South Africa is of great concern.^6,37^ This work is the first known study to identify South Africa districts with higher than expected or, in some instances, rising proportions of patients with AHD that would have been otherwise masked by only examining provincial trends. In 2023, despite a national median exceeding 460 cells/µL, there were three districts where the median CD4 was below 350 cells/µL. The decline in median CD4 values in these districts warrants special programmatic investigations. Of interest was that by 2023, the Sekhukune district reported a percentage of specimens with a count ≤ 200 cells/µL above 30%, yet the rate per 100 000 population had declined substantially. There was a notable reduction in the absolute number of specimens with a count ≤ 200 cells/µL between 2013 and 2023, with a corresponding increase of 13.3% in population estimates.^30^ These findings confirm that, in addition to the percentage of specimens and the rate per population, the absolute number of specimens with a count ≤ 200 cells/µL is an important factor to consider in programmatic interventions. The increase in the median CD4 is also reflected as a continued increase in the percentage of specimens with a count ≥ 500 cells/µL (associated with HIV wellness).^8,16^

The analysis of the 29 districts that reported a percentage point increase in specimens with a count ≤ 200 cells/µL between 2013 and 2023 indicates that over a third of specimens originate from three metropolitan districts. Furthermore, the data outcomes reported here could enable policymakers and programme stakeholders at provincial level to optimise allocation of public funds to target 95-95-95 interventions towards specifically identified populations at the district level with a higher burden of AHD.^38^ However, focusing only on districts with high numbers of specimens with a count ≤ 200 cells/µL would be a challenge in terms of universal coverage.^38^

### Limitations

The laboratory data used in this study could not distinguish between first-ever and follow-up CD4 tests, and may account for differences in disease burden previously reported.^7^ The absence of a unique patient identifier in the public healthcare system in South Africa makes it difficult to de-duplicate data, apart from data repositories built in one province that integrated clinical and laboratory data.^39,40^ There is a need to move towards a person-centred electronic medical record system that supports longitudinal analysis using a unique patient identifier.^40,41^ The absence of clinical data is a limitation. The value of combining laboratory and clinical data systems and socio-economic indicators (i.e. data reported by the Profile and Analysis District Development Model from the Department of Cooperative Governance and Traditional Affairs) to make these programmatic decisions cannot be overemphasised, given the value demonstrated by the Western Cape data centre.^39,42^ There is a need to integrate health data to provide real-time monitoring of the interventions proposed.

## Conclusion

Despite a consistent increase in the national median CD4 count since 2013, persisting high levels of specimens with counts ≤ 200 cells/µL continue to be seen at specific health districts in 2023 that include the Western Cape (Cape Winelands, Overberg, and Garden Route), Eastern Cape (Sarah Baartman, and Nelson Mandela Bay), Gauteng (Ekurhuleni, and Sedibeng) and Limpopo (Sekhukhune) provinces. This warrants urgent attention and further investigation. To our knowledge, the is the first South African study to document the variation in the percentage of specimens with counts ≤ 200 cells/µL at the district level, with an earlier study reporting variation at the provincial level.^8^ Despite lack of de-duplication, this analysis provides important information about specific districts and highlights areas of concern with rising burden of specimens with counts ≤ 200 cells/µL that would otherwise be masked by provincial-level data.^8^ This work provides the impetus for the development of better monitoring systems, including the integration of laboratory and clinical data for meaningful analysis and interpretation at the patient-level to provide a framework for patient-centric data approaches for interventions.
